# Superior growth performance in carp fry achieved with chitosan-alginate encapsulated A-ghrelin versus free peptide: Evidence from physiological, molecular, and morphological analyses

**DOI:** 10.1371/journal.pone.0327235

**Published:** 2025-06-30

**Authors:** Mohammad Matinfar, Behrooz Heidari, AbdolMajid Valipour, Tahereh Rahdari, Sorour Ramezanpour, Mahvash Hadavi, S. Mohsen Asghari

**Affiliations:** 1 Department of Biology, Faculty of Science, University of Guilan, Rasht, Iran; 2 Institute of Biochemistry and Biophysics, University of Tehran, Tehran, Iran; 3 Peptide Chemistry Research Center, K. N. Toosi University of Technology, Tehran, Iran; Benha University, EGYPT

## Abstract

This study investigates the potential of delivering ghrelin via chitosan-alginate (Cs-Al) nanocapsules to enhance growth performance, immune function, and stress biomarker parameters in common carp fry (*Cyprinus carpio*). Characterization of the nanoparticles revealed an average size of 130 nm for Cs-Al particles and 150 nm for A-ghrelin-loaded nanoparticles, with polydispersity indices (PDI) of 0.3 and 0.4, respectively. The zeta potential was measured at +20 mV for Cs-Al nanoparticles and +22 mV for ghrelin-loaded nanoparticles. Morphological analysis using FE-SEM and AFM revealed that the nanoparticles exhibit a predominantly spherical and uniform morphology, while FTIR analysis confirmed their successful synthesis and functionalization. The A-Ghrelin-Cs-Al NP group showed superior growth performance, with higher weight gain vs. control (P < 0.05) and thicker intestinal walls (P < 0.05). While all ghrelin treatments elevated IGF-1 expression (P < 0.05 vs. control), the Cs-Al NP delivery system reduced IgM levels (P < 0.05) and C3 (P < 0.05) compared to free A-Ghrelin, suggesting encapsulation may modulate immune responses. No significant differences in gill morphology were observed between groups (P > 0.05). These findings highlight the potential of ghrelin, particularly when delivered through alginate-chitosan nanocapsules, as a promising tool to promote growth and physiological health in juvenile fish. This approach offers a novel strategy to enhance aquaculture productivity and sustainability through nanotechnology-based delivery systems.

## Introduction

Growth hormone (GH) plays a pivotal role in regulating growth, development, and metabolic processes in vertebrates, including fish. It influences a wide range of physiological functions, such as immune response, osmotic regulation, reproduction, and behavior [1]. The release of GH is primarily controlled by hypothalamic factors, including growth hormone-releasing hormone (GHRH) and ghrelin, a peptide hormone that acts as a potent stimulator of GH secretion [2,3]. Ghrelin, originally identified as an endogenous ligand for the growth hormone secretagogue receptor (GHS-R1a), not only stimulates GH release but also regulates appetite, energy balance, and immune function [4,5].

In aquaculture, the application of bioactive peptides such as ghrelin has emerged as a promising strategy to enhance growth, survival, and immune responses in fish. Goldfish ghrelin12, a synthetic peptide (GTSFLSPAQKPQ), mimics the action of growth hormone-stimulating hormone by binding to GHS-R1a and stimulating GH release from the pituitary gland [6]. This peptide has been shown to improve growth performance, feed efficiency, and protein synthesis in various fish species, including tilapia (*Oreochromis* sp.) and gilthead sea bream (*Sparus aurata*) [7,8]. Furthermore, ghrelin has demonstrated immunomodulatory effects, enhancing antimicrobial activity and non-specific immune responses in fish [9].

Despite its potential, the practical application of ghrelin in aquaculture is limited by its rapid degradation and short half-life *in vivo*. Ghrelin, like many peptide hormones, is susceptible to enzymatic degradation in the gastrointestinal tract, which significantly reduces its bioavailability when administered orally [10]. Additionally, the acidic environment of the stomach can further destabilize the peptide, leading to loss of biological activity. To overcome these challenges, strategies to enhance the molecular stability of ghrelin are essential. Encapsulation within biocompatible and biodegradable polymers, such as alginate and chitosan, has been shown to protect peptides from enzymatic and acidic degradation, thereby improving their stability and controlled release [11]. These delivery systems not only enhance the bioavailability of ghrelin but also ensure sustained release, prolonging its biological activity and efficacy in promoting growth and immune function.

The sustainable growth and health of fish populations are critical for ensuring global food security and economic stability, particularly in the context of increasing demand for aquaculture products. Common carp (*Cyprinus carpio*) is a robust model for aquaculture research, demonstrating responsiveness to dietary bioactive compounds. Studies show carp benefit from immunostimulants like garlic (reducing ammonia toxicity) [12], lavender extract (enhancing innate immunity) [13], and synbiotics (improving disease resistance) [14]. These findings support its suitability for evaluating ghrelin-loaded nanoparticles. However, there is a paucity of research on the use of ghrelin and its encapsulated forms in enhancing the growth and immune performance of carp fry. This study aims to address this gap by investigating the effects of ghrelin, delivered via chitosan-alginate nanocapsules, on growth performance, stress parameters, and immune responses in common carp fry.

The primary objectives of this study are to: (1) evaluate the growth-promoting effects and stability of A-Ghrelin and its encapsulated forms in carp fry, (2) assess the impact of A-Ghrelin on key stress and immune parameters, and (3) explore the potential of chitosan-alginate nanocapsules as a delivery system for sustained release of A-Ghrelin. By elucidating the mechanisms underlying A-Ghrelin’s effects on growth and immunity, this research seeks to provide a foundation for the development of innovative strategies to enhance aquaculture productivity and sustainability.

## Materials and methods

### Ethical statement

All experimental procedures involving animals were conducted in accordance with the ethical guidelines for the care and use of laboratory animals. The study protocol was approved by the Ethics Committee of the University of Guilan (Approval No. IR.GUILAN.REC.1401.013). Efforts were made to minimize stress and discomfort to the animals during handling, sampling, and experimental procedures.

### A-Ghrelin synthesis

The A-Ghrelin peptide (AcGTSFLSPAQKPQ) was synthesized using the standard Fmoc-based solid-phase peptide synthesis (SPPS) protocol on CTC resin. Fmoc-protected amino acids were obtained from commercial sources (Iris Biotech). Fmoc groups were removed using 25% piperidine in DMF. After chain assembly, N-terminal acetylation was performed using acetic anhydride and DIPEA in DMF. The peptide was cleaved from the resin using TFA/TIS/H₂O (93:2:5, v/v/v), precipitated in cold ether, and purified by preparative RP-HPLC (Knauer, Germany) on a C18 column. The final peptide was lyophilized and stored at –20°C. Peptide purity was confirmed by analytical HPLC, and the identity was verified via ESI-MS (Shimadzu LCMS2010A, positive-ion mode; m/z 100–2000).

### Preparation of Chitosan-Alginate (Cs-Al) nanoparticles and A-ghrelin load Chitosan-Alginate (Cs-Al) nanoparticles

Chitosan nanoparticles were prepared using an ionic gelation method [10]. Briefly, 1 mg of chitosan was dissolved in 1 mL of 1% (v/v) acetic acid (pH 5.0), and 200 µg of A-Ghrelin (synthesized via Fmoc-based solid-phase peptide synthesis) was added to the solution. Sodium tripolyphosphate (TPP, 1 mg/mL) was used as a crosslinking agent, and nanoparticles were formed by dropwise addition of 2 mL of TPP solution to 5 mL of the chitosan- A-Ghrelin mixture under continuous magnetic stirring at room temperature for 2 h. The resulting nanoparticles were then encapsulated in alginate by adding the chitosan- A-Ghrelin solution dropwise to 20 mL of 0.45 mg/mL alginate solution (pH 5.0). The mixture was sonicated for 15 min, followed by the addition of 4 mL of 0.67 mg/mL CaCl_2_ solution to induce gelation. The final nanocapsules were collected and freeze-dried for further use.

### Synthesis and characterizations of nanoparticles

#### Nanoparticle size and Zeta Potential.

The average size and zeta potential of the nanocapsules were assessed with a Zetasizer from Malvern Instruments in England. Dynamic light scattering (DLS) was employed to determine the average size of the nanoparticles. The zeta potential of pre- and post- A-Ghrelin encapsulation was evaluated by measuring the electrophoretic mobility of the particles in an aqueous suspension.

#### Absorption characteristics and calibration curve of A-Ghrelin.

The calibration curve for A-Ghrelin was generated by measuring the UV-Vis absorption spectra of known concentrations of the peptide (ranging from 2 to 30 µg/mL) over the wavelength range of 200–350 nm. As shown in the attached figure, A-Ghrelin exhibits two primary absorption peaks: one at approximately 230 nm (attributable to peptide bonds) and another near 280 nm (due to aromatic residues). For quantification purposes, we selected the absorbance at 230 nm to construct a linear regression curve correlating absorbance with concentration.

#### Encapsulation efficiency.

The encapsulation efficiency (EE%) of A-Ghrelin in Cs-Al nanoparticles was calculated via UV-Vis spectrophotometry. The free peptide was separated by centrifugation, and its concentration was compared against the calibration curve to derive EE%.


$$\text{Encapsulation\textbackslash{} Efficiency }(\;\%)=\frac{Total\;ghrelin\;added\;during\;the\;synthesis-Free\;ghrelin\;in\;suspernatant}{Total\;ghrelin\;added\;during\;the\;synthesis}\times 100$$

#### Stability of A-Ghrelin-loaded nanoparticles.

To assess the environmental stability of the A-Ghrelin-loaded nanoparticles, the freeze-dried powder was stored at −20°C for one month. Subsequently, the particle size, polydispersity index (PDI), and surface charge were analyzed using dynamic light scattering (DLS) [15].

#### FTIR analysis and morphological study.

The synthesis accuracy of nanoparticles and A-Ghrelin-loaded nanoparticles was confirmed by evaluating their functional groups using Fourier Transform Infrared Spectroscopy (FTIR) [16]. In addition, the morphology of both the nanoparticles and A-Ghrelin-loaded nanoparticles was analyzed using Field Emission Scanning Electron Microscopy (FE-SEM) and atomic force microscopy (AFM) [17].

### In Vitro A-Ghrelin release from nanoparticles

The A-Ghrelin loaded Cs-Al nanoparticles (1 mg/mL) were placed inside a dialysis bag, which was then floated in 30 mL of external phosphate buffer and positioned on a heater-stirrer. The release study was performed at 25°C with constant stirring (100 rpm) to maintain nanoparticle suspension and simulate fluid dynamics in the fish digestive tract. The release was initially evaluated at neutral pH. In a separate experiment, the solution was exposed to acidic gastric pH (pH 3) for 3 hours, followed by a transition to alkaline intestinal pH (pH 8.5) for 3 hours. During this process, 1 mL of the external solution was withdrawn every 15 minutes, and fresh buffer was added to maintain a constant external volume. The absorbance of the collected samples was then measured, and the concentration of the released A-Ghrelin was calculated over time.

### Experimental design, dosage optimization, and feeding trial

A total of 150 common carp fry (*Cyprinus carpio*; initial weight: 0.8 ± 0.08 g; length: 2.65 ± 0.53 cm) were acclimated for two weeks in a 500-L tank with controlled conditions (pH: 7.6 ± 0.2; temperature: 26 ± 1°C; dissolved oxygen: 6.4 ± 0.1 mg/L) while fed a commercial diet (Gilak Daneh Navid, Rasht, Iran) (2% body weight/day). To determine the optimal A-Ghrelin dosage, fry (30 fish/treatment and n = 10/replicate) were fed diets containing 0, 50, 100, 200, or 400 µg/kg A-Ghrelin for four weeks, with 100 µg/kg identified as optimal based on growth performance and *igf1* expression. For the main experiment, 180 fry were divided into six groups (30 fish/treatment, n = 10/replicate): (1) **Control**: Negative control that fed a diet containing phosphate-buffered saline (PBS), (2) **A-Ghrelin**: Fed a diet containing 100 µg/kg free A-Ghrelin, (3) **A-Gherlin-loaded Cs-Al**: Fed a diet containing A-Ghrelin-loaded chitosan-alginate nanoparticles, (4) **A-Ghrelin Loaded Cs**: Fed a diet containing A-Ghrelin-loaded chitosan nanoparticles, (5) **Cs-Al + A-Ghrelin**: Fed a diet containing unloaded chitosan-alginate mixed with free A-Ghrelin, and (6) **Cs-Al**: Fed a diet containing unloaded chitosan-alginate nanoparticles. Fish were fed five times daily to satiety for four weeks, with water quality (pH, temperature, dissolved oxygen) monitored daily using a multiparameter probe (YSI ProDSS, USA) and 30% water replacement every three days.

### Sampling and analysis

At the end of the experiment, fish were anesthetized with clove extract (20 mg/L) and euthanasia was performed by destroying the brain with a sterile scalpel blade, following ethical guidelines [18]. Liver tissue was collected for *igf1* gene expression analysis using quantitative real-time PCR (qRT-PCR). The whole fish body was homogenized, and the homogenate was diluted 1:2 with PBS (pH 7.2) and analyzed for growth hormone, cortisol, glucose, total protein, and immune parameters (IgM, C3, and lysozyme). Histological examinations of the intestine and gills were performed using hematoxylin and eosin (H&E) staining.

### Relative expression of the insulin-like growth factor 1 (*igf1*) gene

After dissecting the fish, the liver tissue was utilized for gene expression analysis. RNA extraction was performed using the TRIzol method according to the manufacturer’s instructions. The RNA concentration and purity were determined using the NanoDrop spectrophotometer technique (Thermo Scientific). The RNA samples were measured for their absorbance at wavelengths of 260 nm and 280 nm to ascertain both the concentration and the absorbance ratio. A desirable range for this ratio is typically between 1.8 and 2.1, indicating high-quality RNA free from contamination by proteins, phenols, or other impurities. Subsequently, for the conversion of the extracted RNA into complementary DNA (cDNA), a Thermo Scientific Kit (Thermo Fisher Scientific, Cat. No. 18091050) was employed following the manufacturer’s instructions.

To design primers for the *igf1* gene in common carp (*Cyprinus carpio*), the nucleotide sequence was retrieved from gene databases accessible at www.NCBI.NIH.gov/GeneBank. Using GeneRunner software, a specific segment was identified, and unique forward and reverse primers were designed for the *igf1* gene (Table 1). The reference gene used in this study was β-actin.

**Table 1:** The primer sequences for the insulin-like growth factor 1 (*igf1*) and β-actin genes of common carp (*Cyprinus carpio*).

Gene	Direction	Sequence (5’ → 3’)	Amplicon Size (bp)	Accession Number
*Igf1*	Forward	CGCCTCGAGATGTATTGTGCAC	148	XM_019096355.1
	Reverse	CTGTATGCCGTTGCGCTCGT		
β-actin	Forward	GACTTCGAGCAGGAGATGGG	122	AB039726.1
	Reverse	CCGCAAGATTCCATACCCAGG		

Real-time quantitative PCR (qRT-PCR) was conducted using the SinaClon kit (Iran) on a light cycler 96 machine (Roche, Germany). The qRT-PCR reactions were performed in triplicate with a total reaction volume of 25 μL. The qRT-PCR program consisted of an initial denaturation step at 95°C for 15 minutes, followed by three amplification steps at 45 cycles: 30 seconds at 90°C, 30 seconds at 61°C, and 10 minutes at 72°C. The final step involved lowering the temperature to 4°C.

The quantification cycle (Ct) values were determined using the light cycler 961.1 application software provided by Roche, Germany. The 2^-(ΔΔCt)^ method was employed to calculate the relative changes in *igf1* gene expression levels compared to β-actin, providing insights into the regulation of gene expression in common carp.

### Histology

For histological studies, samples of the midsection of the intestine and gills were fixed in 10% formalin. Following the fixation process, the tissues underwent a series of washes using 70% ethanol to remove residual fixative and prepare them for subsequent processing steps. Subsequently, the tissue processing continued with sequential clearing and additional dehydration steps involving a gradual transition from ethanol to xylene concentrations. Upon successful preparation of paraffin blocks from the tissue samples, precision cutting using a microtome was employed to obtain thin sections (5–6 µ). The sections were subjected to staining with the classic hematoxylin and eosin (H&E) technique [19].

### Immune parameter analysis

Serum immune parameters were quantified using standardized assays: IgM levels and complement C3 were measured by ELISA (Hangzhou Eastbiopharm kits) following manufacturer protocols, with absorbance read at 450 nm after streptavidin-HRP/TMB development [20]. Lysozyme activity was determined turbidimetrically by mixing 25 μL serum with 175 μL Micrococcus lysodeikticus suspension (0.2 mg/mL in PBS, pH 6.4) and monitoring the ΔA450/min (1 unit = 0.001 absorbance decrease/min) [21]. All assays included triplicate measurements, appropriate standards and blanks.

### Determination of growth hormone

The detection and quantification of growth hormone, were facilitated through the employment of specialized ELISA kits. The growth hormone ELISA kit (Product Code: CSB-E12121Fh) utilized in the study was obtained from CUSABIO, a trusted supplier recognized for its innovative immunoassay products. This kit functioned by binding antibodies to specific antigen targets, thereby generating measurable signals directly proportional to the quantity of analytes present in the samples.

### Stress parameters

Stress-related biochemical parameters were analyzed using established methods: cortisol levels were quantified via competitive ELISA (Monobind Inc., Cat 300–3625) following manufacturer protocols, while glucose concentrations were determined using the ortho-toluidine method, where 50 μL serum samples reacted with 2 mL O-toluidine reagent at 100°C for 10 minutes before absorbance measurement at 630 nm. Both assays were performed in triplicate with appropriate standard curves [22].

### Alanine Aminotransferase (ALT) and Aspartate Aminotransferase (AST)

According to [23], ALT and AST activities were estimated using commercially accessible Pars Azmoon diagnostic kits. Both enzymatic activities were assessed employing a colorimetric method at a wavelength of 340 nm alongside maintaining a constant temperature of 37°C. Notably, the minimum sensitivity level for these kits stood at 2 units per liter (U/L).

### Total protein

3 g of copper sulfate pentahydrate (CuSO_4_.5H_2_O) and 9 g of sodium potassium tartrate were dissolved in 500 ml of 0.2 mol/l sodium hydroxide; 5 g of potassium iodide was added and made up to 1 liter with 0.2 mol/l sodium hydroxide (Biuret Reagent). 0.0, 0.2, 0.4, 0.6, 0.8, and 1 mL of working standard (Bovine serum albumin) were pipetted out into labeled test tubes. 1 mL of the given sample was pipetted out into another test tube. The volume was made up to 1 mL in all the test tubes. A tube with 1 mL of distilled water served as the blank. 3 mL of Biuret reagent was added to all the test tubes. The tubes were shaken and warmed at 37 ºC for 10 min. The contents were cooled to room temperature, and the absorbance at 540 nm against the blank was recorded

### Growth indices

During the experimental period, the fry were individually weighed on the first day and at the end of each week. The daily feed intake was recorded as well. The change in fry weight was calculated using Formula 1: weight gain = final weight-initial weight. Additionally, the feed conversion ratio (FCR), the specific growth rate (SGR) and condition factor (K) were calculated using Formulas 2, 3 and 4, respectively.

Formula 2: FCR = average feed intake/average weight gain

Formula 3: SGR = [(Ln final weight - Ln initial weight)/ days] × 100.

Formula 4: K = (body mass/ (body length)^3^) × 100.

### Statistical analysis

Data were analyzed using one-way ANOVA followed by Duncan’s post-hoc test (SPSS v19.0), with the tank (not individual fish) as the experimental unit. Normality was verified using Kolmogorov-Smirnov tests (p > 0.05 for all parameters), and homogeneity of variance was confirmed with Levene’s test. Results are presented as mean ± standard deviation (SD), with statistical significance set at p < 0.05. 30 fish in each treatment were sampled.

## Results

### Synthesis and characterization of nanoparticles

#### Size and Zeta potential.

**Fig 1:**
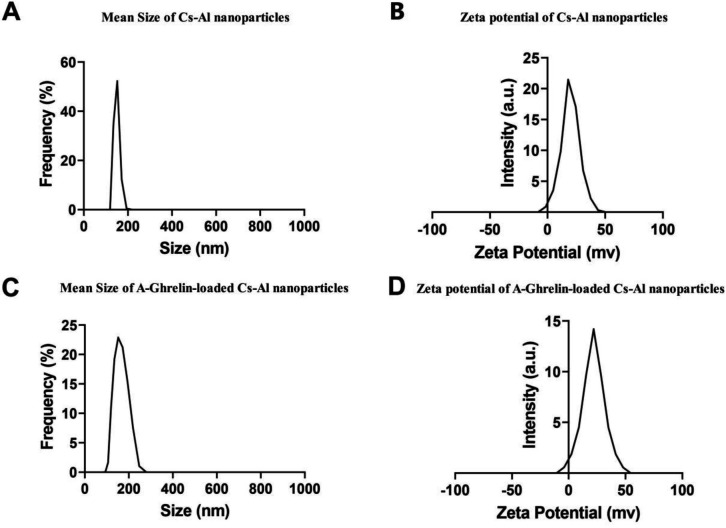
Size, PDI and zeta potential of nanoparticles analyzed by DLS. A: Mean size and PDI of Cs-Al nanoparticles (130 nm, PDI = 0.3). B: Zeta potential of Cs-Al nanoparticles (20 mV). C: Mean size and PDI of A-Ghrelin loaded Cs-Al nanoparticles (150 nm, PDI = 0.4). D: Zeta potential of Cs-Al ghrelin-loaded nanoparticles (22mV).

Fig 1A,1B) presents an average nanoparticle size of 130 nm and a polydispersity index (PDI) of 0.3, indicating uniformity and an appropriate size for the nanocarrier. The zeta potential of the chitosan-alginate (Cs-Al) nanoparticles was measured at 20 mV. For the A-Ghrelin-loaded nanoparticles, the mean size increased to 150 nm, with a PDI of 0.4 and a zeta potential of 22 mV (Fig 1C,1D). These findings indicate that the nanoparticles maintained stability following peptide loading. After one month, no significant changes were observed in the size, dispersion index, or surface charge of the A-Ghrelin-loaded nanoparticles, indicating their environmental stability (data not shown).

#### Morphological study of nanoparticles.

**Fig 2:**
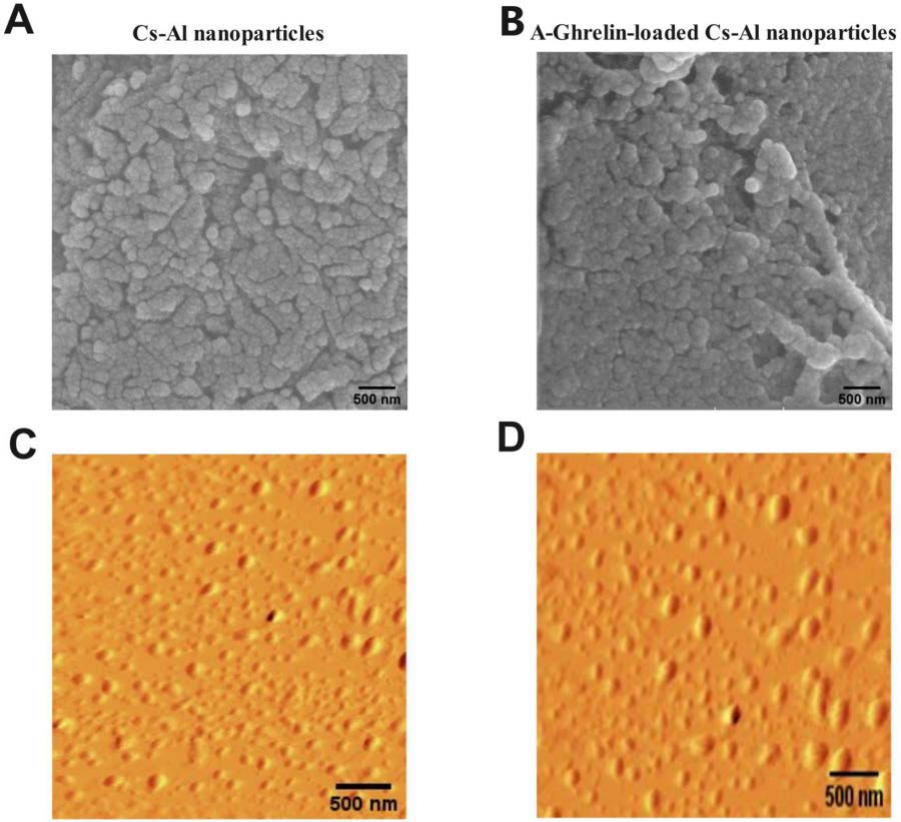
Morphology of nanoparticles. A: Morphological analysis of Cs-Al nanoparticles by FE-SEM. B: Morphological analysis of A-Ghrelin loaded-nanoparticles by FE-SEM. C: Morphological analysis of Cs-Al nanoparticles by AFM. D: Morphological analysis of A-Ghrelin-loaded nanoparticles by AFM. Scale bar: 500 nm.

Analysis of the Cs-Al nanoparticles using Field Emission Scanning Electron Microscopy (FE-SEM) and atomic force microscopy (AFM) revealed a predominantly spherical and uniform morphology. This morphology and uniformity were maintained even after A-Ghrelin loading (Fig 2A-2D).

#### Encapsulation efficiency.

The encapsulation efficiency of the A-Ghrelin in chitosan-alginate nanoparticles was found to be 73.8%.

#### FTIR Analysis.

**Fig 3:**
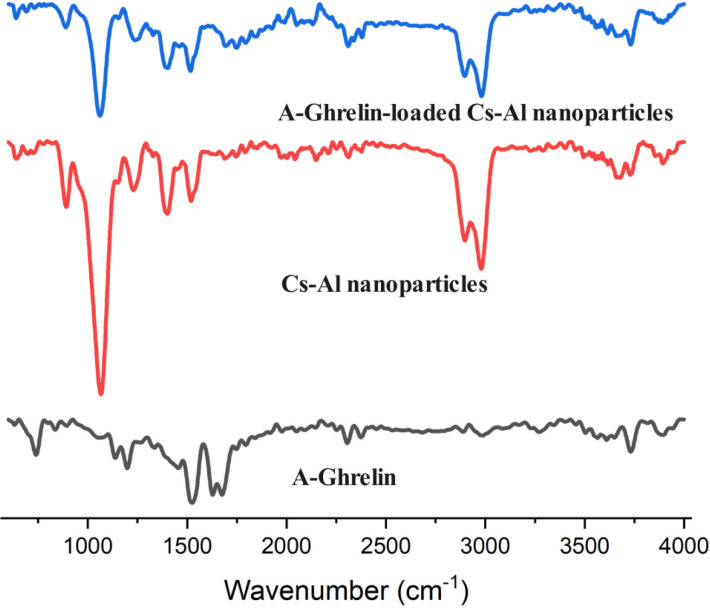
FTIR spectrum of A-Ghrelin (black line), Cs-Al nanoparticles (red line) and A-Ghrelin-loaded Cs-Al nanoparticles (blue line). The FTIR spectra confirm the successful incorporation of A-Ghrelin into the Cs-Al nanoparticle matrix.

The bands at 3678 cm^−1^ and 13893 cm^−1^ correspond to the stretching of –OH and –NH2, indicating the presence of amino and hydroxyl groups in chitosan. The bands at 1510 and 1400 cm^−1^ are associated with the carboxylic acid group in sodium alginate (Fig 3). Specific peaks for the A-Ghrelin include the -NH stretching band in the 1511 cm^−1^ range and the C = O stretching band in the 1645 cm^−1^ range, indicating amide and carbonyl bonds. Additionally, peaks between 1675–1455 cm^−1^ are linked to vibrations from aromatic rings. The disappearance of A-Ghrelin peaks in the A-Ghrelin -loaded Cs-Al nanoparticles indicated complete entrapment within the matrix (Fig 3). This observation is consistent with previous studies where successful drug entrapment led to the disappearance of drug-specific FTIR peaks [24–29].

#### A-Ghrelin release from Cs-Al nanoparticles.

**Fig 4:**
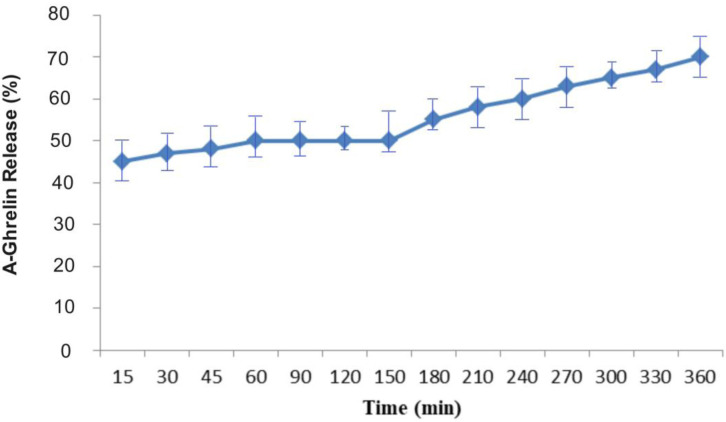
A-Ghrelin release in acidic and then basic conditions. The release profile indicates that, overall, 70% of A-Ghrelin was released over a period of 360 minutes. Approximately 50% of A-Ghrelin was released immediately upon exposure to acidic conditions (pH 3) during the first 180 minutes. After 180 minutes in a basic pH environment (pH 8.5), an additional 20% of A-Ghrelin was gradually released.

The in vitro release study demonstrated a pH-dependent release profile of A-Ghrelin from the Cs-Al nanoparticles. Under acidic conditions (pH 3.0, simulating gastric environment), approximately 50% of the encapsulated A-Ghrelin was rapidly released within the first 180 minutes, likely due to protonation-induced swelling of the polymeric matrix. Subsequently, when the pH was adjusted to 8.5 (simulating intestinal conditions), an additional 20% of A-Ghrelin was gradually released over the following 180 minutes, resulting in a cumulative release of 70% over the total 360-minute study period (Fig 4).

### Optimal dosage determination

**Fig 5:**
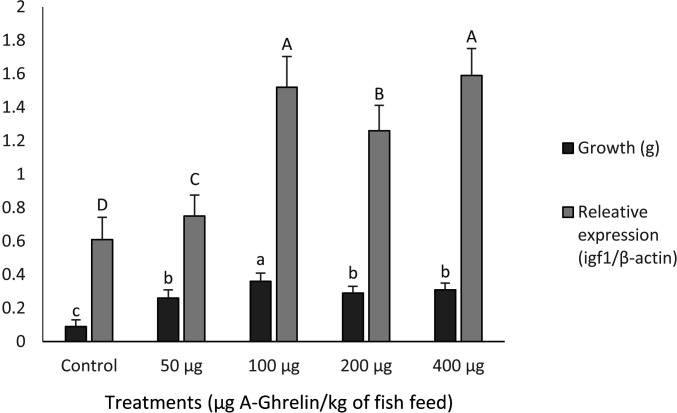
Dose-response effects of A-Ghrelin on *igf1* gene expression and growth performance in common carp fry. Different letters denote significant differences (P < 0.05).

Significant dose-dependent effects on *igf1* gene expression and fry growth were observed (P < 0.0001). While both 100 μg/kg and 400 μg/kg doses showed comparable effects on *igf1* expression (Fig 5), the 100 μg/kg dose was selected as optimal because the highest weight gain were achieved at a dosage of 100 µg A-Ghrelin per kilogram of feed and offered substantial cost advantages for ghrelin production.

### Immune parameters

**Fig 6:**
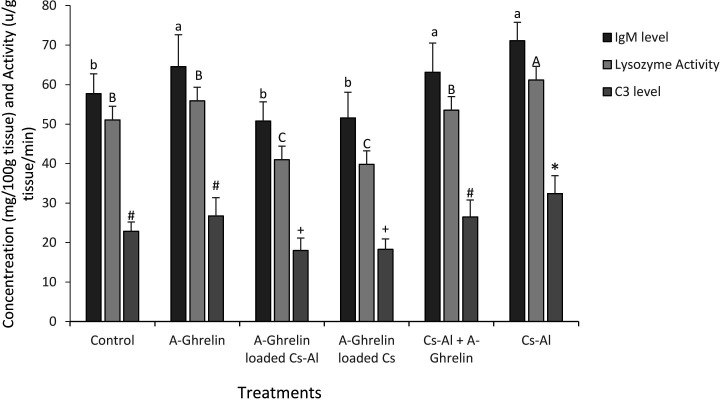
Effects of A-Ghrelin treatments on immune parameters (IgM, C3, and lysozyme activity) in common carp fry. Different letters indicate significant differences (P < 0.05). The term “Cs” likely refers to chitosan, while “Cs-Al” denotes the composite of chitosan-alginate. The A-Ghrelin group was given only acetylated goldfish Ghrelin with feed, A-Gherlin-loaded Cs-Al received acetylated goldfish Ghrelin-infused chitosan-alginate nanoparticles with feed, A-Ghrelin loaded Cs received chitosan nanoparticle-encapsulated A-Ghrelin with feed, and Cs-Al + A-Ghrelin unloaded chitosan-alginate in conjunction with A-Ghrelin in feed. The negative control received PBS with feed and the positive control received chitosan-alginate nanoparticles with feed.

A-Ghrelin treatments did not significantly alter IgM, C3, or lysozyme activity compared to controls (P < 0.0001). The Cs-Al group exhibited the highest immune marker levels, while A-Ghrelin loaded Cs-Al and A-Ghrelin loaded Cs showed the lowest (Fig 6).

### Stress parameters

**Fig 7:**
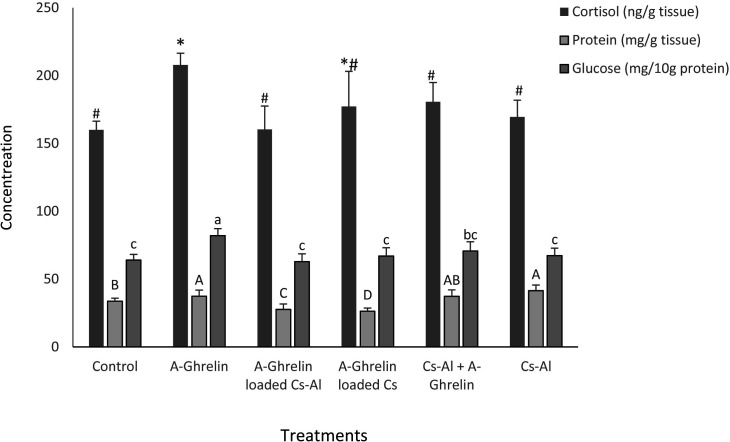
Changes in total protein, cortisol, and glucose levels in response to A-Ghrelin treatments. Treatment groups and statistical notations follow the same conventions as described in Fig 6.

A-Ghrelin treatment significantly elevated cortisol and glucose levels (P < 0.0001), while total protein levels were highest in the A-Ghrelin group and lowest in A-Ghrelin loaded Cs-Al (P < 0.0001) (Fig 7).

### Growth Hormone and *igf1* Gene expression

**Fig 8:**
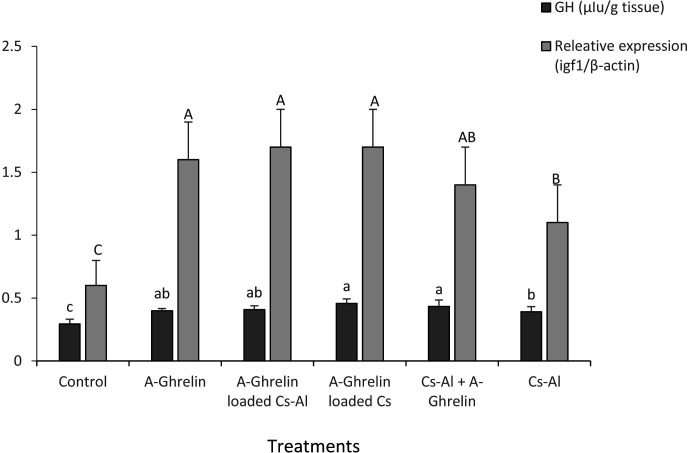
Effects of A-Ghrelin on growth hormone levels and *igf1* gene expression. Treatment groups and statistical notations follow the same conventions as described in Fig 6.

A-Ghrelin treatments significantly increased growth hormone (GH) levels and *igf1* gene expression compared to controls (P < 0.0001). No significant differences were observed among A-Ghrelin, A-Ghrelin loaded Cs-Al, A-Ghrelin loaded Cs, and Cs-Al+ghrelin treatments (Fig 8).

### Hepatic Enzyme activity

**Fig 9:**
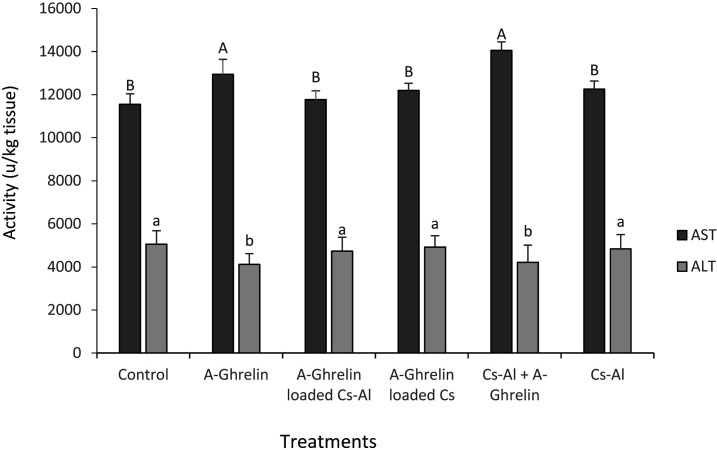
Changes in ALT and AST activity in response to A-Ghrelin treatments. Treatment groups and statistical notations follow the same conventions as described in Fig 6.

A-Ghrelin treatment significantly altered ALT and AST activity (P < 0.0001). The highest AST activity and lowest ALT levels were observed in the A-Ghrelin and Cs-Al + A-Ghrelin groups (Fig 9).

### Weight gain

**Fig 10:**
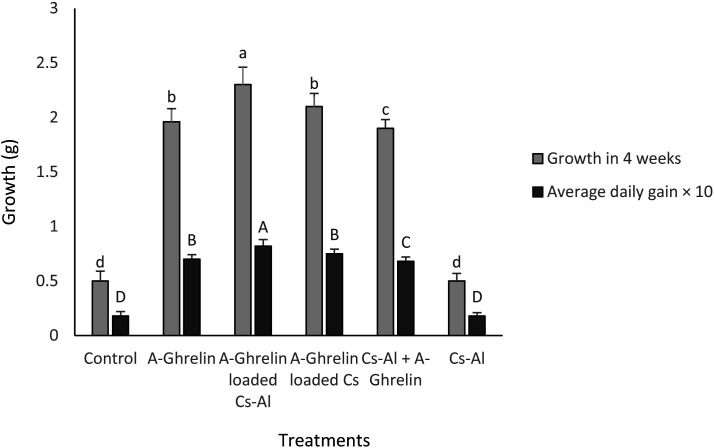
Weight gain in common carp fry across treatment groups. Treatment groups and statistical notations follow the same conventions as described in Fig 6.

The A-Ghrelin loaded Cs-Al group exhibited the highest weight gain, with fry weight increasing from 0.5 g to 2.3 g over four weeks (0.018 g to 0.082 g daily) (P < 0.0001) (Fig 10).

### Feed conversion ratio (FCR)

**Fig 11:**
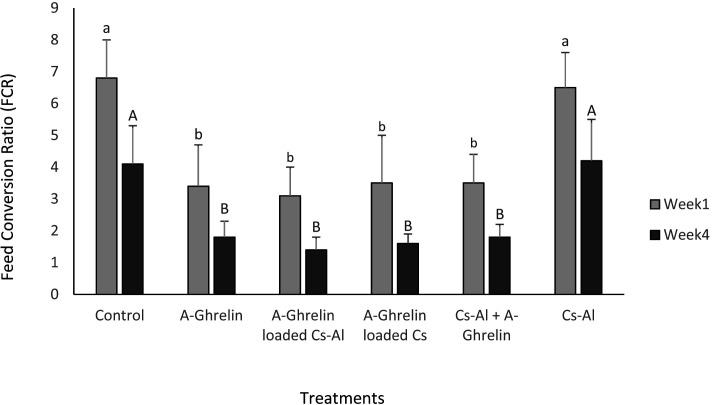
Changes in feed conversion ratio (FCR) across treatment groups. Treatment groups and statistical notations follow the same conventions as described in Fig 6.

The lowest FCR values were recorded in A-Ghrelin, A-Ghrelin loaded Cs-Al, A-Ghrelin loaded Cs, and Cs-Al+ghrelin groups (P < 0.0001). The A-Ghrelin loaded Cs-Al group showed a 2.9-fold reduction in FCR compared to controls (Fig 11).

### Specific Growth Rate (SGR), condition factor and survival rate

**Fig 12:**
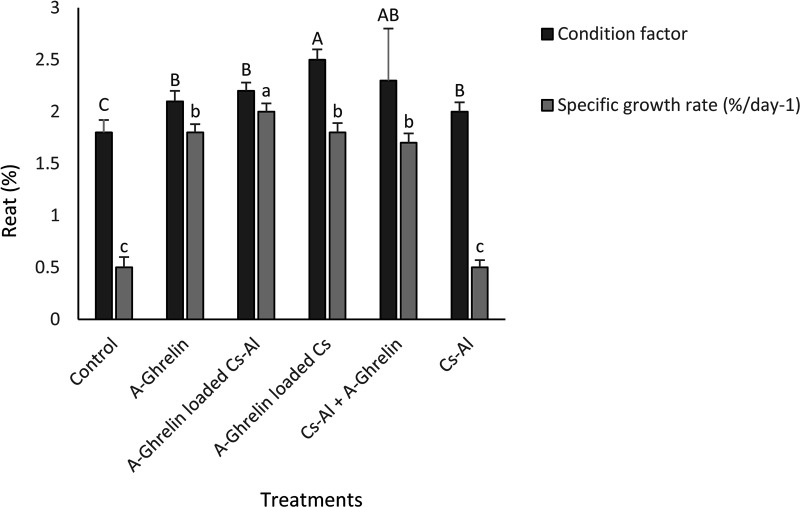
Effects of A-Ghrelin on specific growth rate (SGR) and condition factor. Treatment groups and statistical notations follow the same conventions as described in Fig 6.

A-Ghrelin treatments significantly increased SGR and condition factor (P < 0.0001), with the highest SGR observed in the A-Ghrelin loaded Cs-Al group (Fig 12). In this study, no mortality occurred across all treatment groups during the 4-week trial, resulting in a 100% survival rate.

### Intestinal Morphology

**Fig 13:**
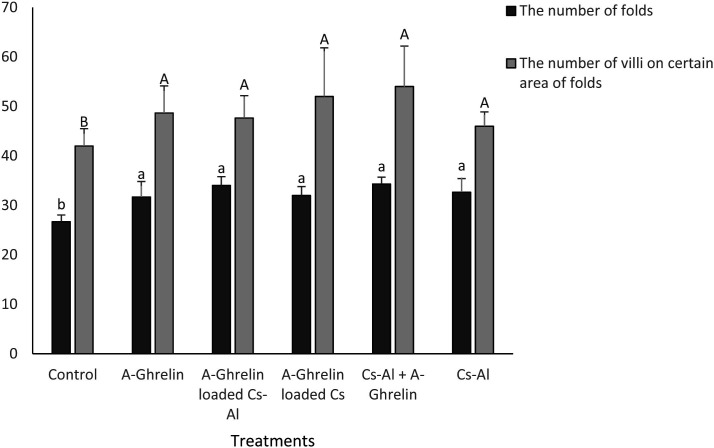
Changes in intestinal fold and villi numbers. The graph shows the average number of folds in the intestines of three fish. The average number of villi corresponds to a specific level of folds in the intestines of these three fish. Each intestine is divided into three sections. Treatment groups and statistical notations follow the same conventions as described in Fig 6.

**Fig 14:**
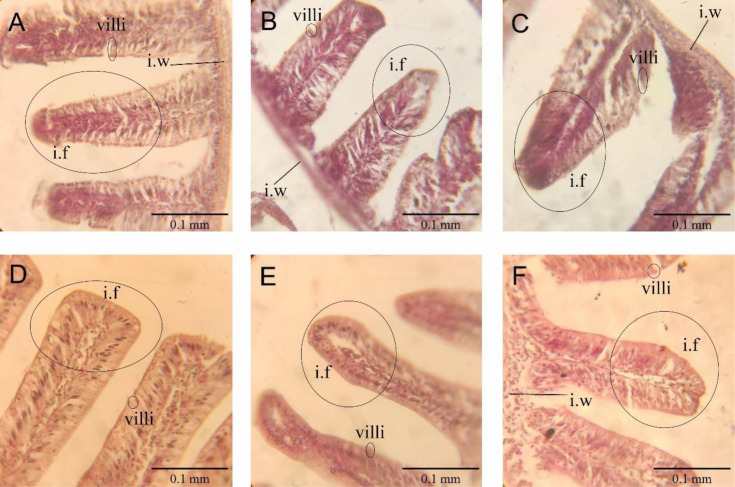
Histological cross-sections of the small intestine (40 × magnification): (A) Control; (B) A-Ghrelin; (C) A-Gherlin-loaded Cs-Al; (D) A-Ghrelin loaded Cs; (E) Cs-Al + A-Ghrelin; (F) Cs-Al. Key structures are labeled: intestinal fold (i.f), villi, and intestinal wall (i.w).

**Fig 15:**
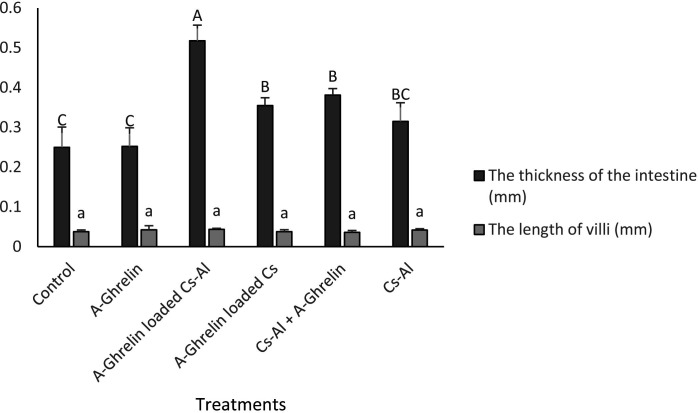
Changes in intestinal wall thickness and villi length. Treatment groups and statistical notations follow the same conventions as described in Fig 6.

A-Ghrelin treatments significantly increased intestinal fold (26.67 to 34 in A-Ghrelin loaded Cs-Al group) and villi numbers (49 to 54 in Cs-Al + A-Ghrelin group) (P < 0.05). The A-Ghrelin loaded Cs-Al group exhibited the greatest intestinal wall thickness (0.518 mm) compared to controls (0.250 mm) (P < 0.0001) (Figs 13–15).

### Histology of gills

**Fig 16:**
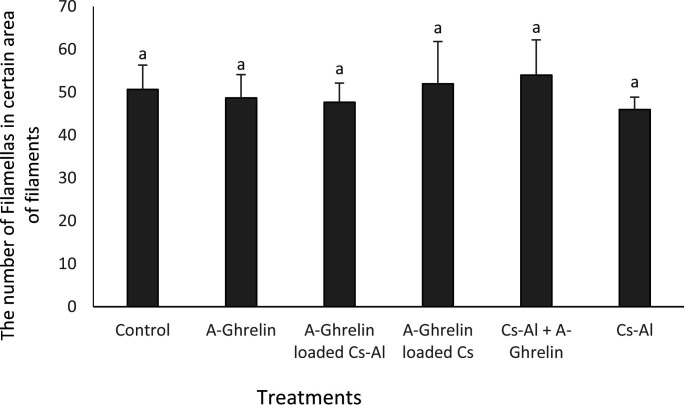
Number of gill lamellae across treatment groups. Treatment groups and statistical notations follow the same conventions as described in Fig 6.

**Fig 17:**
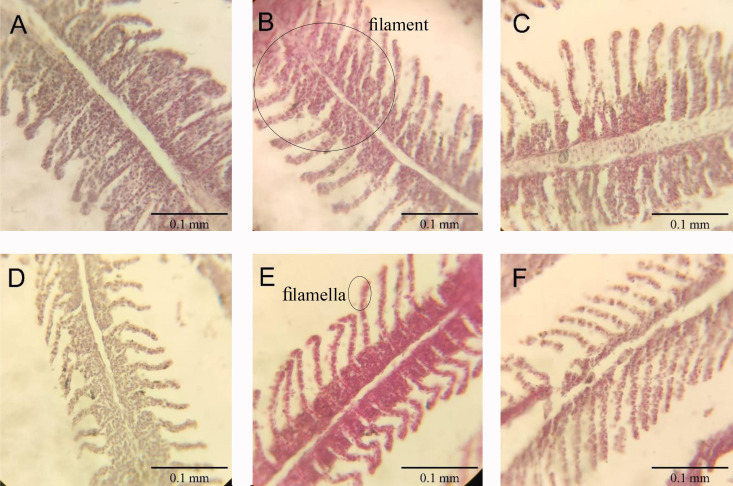
Histological cross-sections of gill (40 × magnification): (A) Control; (B) A-Ghrelin; (C) A-Gherlin-loaded Cs-Al; (D) A-Ghrelin loaded Cs; (E) Cs-Al + A-Ghrelin; (F) Cs-Al.

No significant differences in gill lamellae were observed among treatment groups (P > 0.05) (Fig 16 and 17).

## Discussion

The findings of this study demonstrate that A-Ghrelin, particularly when delivered via chitosan-alginate nanocapsules, significantly enhances growth performance, stress parameters, and immune function in common carp fry. The use of nanotechnology for peptide delivery offers a promising strategy to overcome the limitations of rapid degradation and short half-life associated with A-Ghrelin, thereby improving its bioavailability and efficacy in aquaculture applications.

The successful synthesis of chitosan-alginate nanocapsules was confirmed through morphological analysis, dynamic light scattering (DLS), and FTIR spectroscopy. The nanocapsules exhibited uniform size distribution (130 nm), moderate zeta potential (22 mV), and high encapsulation efficiency (73.8%), ensuring stability and controlled release of A-Ghrelin. The pH-dependent release profile, with 70% of A-Ghrelin released under acidic and basic conditions, aligns with the physiological environment of the fish gastrointestinal tract. This suggests that the nanocapsules protect A-Ghrelin during passage through the stomach, enabling sustained release in the intestine, where absorption occurs. These findings are consistent with previous studies highlighting the potential of chitosan-alginate systems for oral delivery of bioactive compounds in aquatic species [10].

A-Ghrelin treatment significantly increased circulating growth hormone (GH) concentrations and upregulated hepatic *igf1* gene expression, consistent with its established role as a potent endogenous growth hormone secretagogue in teleost fish. The highest *igf1* expression and weight gain were observed at a dosage of 100 µg A-Ghrelin per kilogram of feed, establishing this as the optimal dose. These results are in agreement with studies in tilapia (*Oreochromis* sp.) and gilthead sea bream (*Sparus aurata*), where ghrelin administration enhanced growth performance and feed efficiency [7,30]. The significant increase in total protein levels in ghrelin-treated fry further supports its role in promoting protein synthesis and growth, consistent with the anabolic effects of growth hormone [31].

Ghrelin also induced a marked increase in glucose and cortisol levels, likely due to its role in stimulating gluconeogenesis and glycogenolysis, as well as its interaction with the hypothalamic-pituitary-adrenal axis [32,33]. However, the encapsulated forms of A-Ghrelin did not exhibit the same effects on glucose levels, suggesting that the delivery system may modulate the physiological response. This highlights the need for further investigation into the mechanisms underlying A-Ghrelin’s physiological effects in fish.

While A-Ghrelin treatment did not significantly enhance IgM, C3, or lysozyme activity, the Cs-Al group exhibited the highest levels of these immune markers. These results suggest that the chitosan-alginate nanoparticle matrix exhibits intrinsic immunomodulatory properties that are independent of A-Ghrelin’s biological activity. Previous studies have reported that chitosan and alginate can enhance non-specific immune responses in fish, potentially through their interactions with mucosal surfaces in the gastrointestinal tract [21]. The absence of a significant immune response to ghrelin in common carp contrasts with findings in tilapia, where ghrelin administration enhanced both antimicrobial activity and lymphocyte proliferation [9]. This discrepancy could arise from species-specific variations in immune regulation, differences in ghrelin dosage, or the distinct delivery method (nanocapsule vs. free peptide) employed in this study.

A-Ghrelin treatment altered hepatic enzyme activity, with significant increases in AST and decreases in ALT levels. These changes may reflect enhanced physiological activity in the liver, consistent with the role of A-Ghrelin in promoting growth and nutrient utilization. The encapsulated forms of ghrelin, however, prevented these alterations, likely due to the controlled release of the peptide, which minimizes acute physiological stress [34,35].

Histological analysis revealed that A-Ghrelin treatment significantly increased intestinal fold and villi numbers, as well as intestinal wall thickness. These morphological changes are indicative of improved nutrient absorption, which may contribute to the observed growth enhancement. The A-Ghrelin-loaded Cs-Al group exhibited the greatest intestinal wall thickness, indicating that the chitosan-alginate nanocapsule delivery system enhances the stability and targeted release of A-Ghrelin in the gastrointestinal tract, thereby amplifying its bioactivity. These findings align with studies in shrimp (*Litopenaeus schmitt*), where ghrelin immersion treatments improved growth and intestinal morphology [36].

The use of A-Ghrelin, particularly in encapsulated form, offers a novel approach to enhancing growth and feed efficiency in aquaculture. The significant improvements in specific growth rate (SGR), feed conversion ratio (FCR), and intestinal morphology observed in this study highlight the potential of A-Ghrelin as a growth promoter in juvenile fish. Moreover, the chitosan-alginate delivery system offers a cost-effective and sustainable approach to improve the stability and efficacy of bioactive peptides in aquaculture feeds.

While this study provides valuable insights into the effects of A-Ghrelin on growth and immune function in common carp fry, several limitations should be addressed in future research. First, the mechanisms underlying the stress and immune effects of A-Ghrelin require further elucidation, particularly in relation to species-specific responses. Second, long-term studies are needed to assess the potential effects of A-Ghrelin on reproductive performance and stress tolerance in adult fish. Finally, the economic feasibility of large-scale production and application of A-Ghrelin-loaded nanocapsules should be evaluated to ensure practical implementation in aquaculture.

## Conclusion

In conclusion, this study demonstrates that A-Ghrelin, delivered via chitosan-alginate nanocapsules, significantly enhances growth performance, stress parameters, and intestinal morphology in common carp fry. The findings underscore the potential of nanotechnology-based delivery systems to improve the efficacy of bioactive peptides in aquaculture, contributing to sustainable and efficient fish production. Although the inclusion of ghrelin-loaded nanoparticles slightly increases feed production costs, this is offset by the considerable improvement in feed conversion efficiency and growth performance observed in treated fish. The enhanced growth rate leads to a higher biomass yield over the same production cycle, which ultimately improves overall profitability. On a commercial scale, these benefits are expected to outweigh the additional input costs within a relatively short operational period, making this approach economically viable for aquaculture enterprises. Future research should focus on optimizing delivery methods and exploring the broader applications of A-Ghrelin in aquaculture.

## Supporting information

S1 Fig Absorption characteristics of A-ghrelin via UV-visible spectroscopy. A: UV-Visible Absorption Spectrum of A-ghrelin at Different Concentrations, B: The resulting calibration curve of A-ghrelin illustrates the correlation between absorbance and concentration.
(TIF)

S2 File Raw data values for biochemical and physiological analyses reported in this manuscript. (XLSX)

S3 File Raw data values for histometric and growth indices analyses reported in this manuscript. (XLSX)
