# Supplementary figures and images for: Superior growth performance in carp fry achieved with chitosan-alginate encapsulated A-ghrelin versus free peptide: Evidence from physiological, molecular, and morphological analyses
Source: PLoS One. 2025 Jun 30;20(6):e0327235. doi: 10.1371/journal.pone.0327235 (PMC12208430; doi:10.1371/journal.pone.0327235)

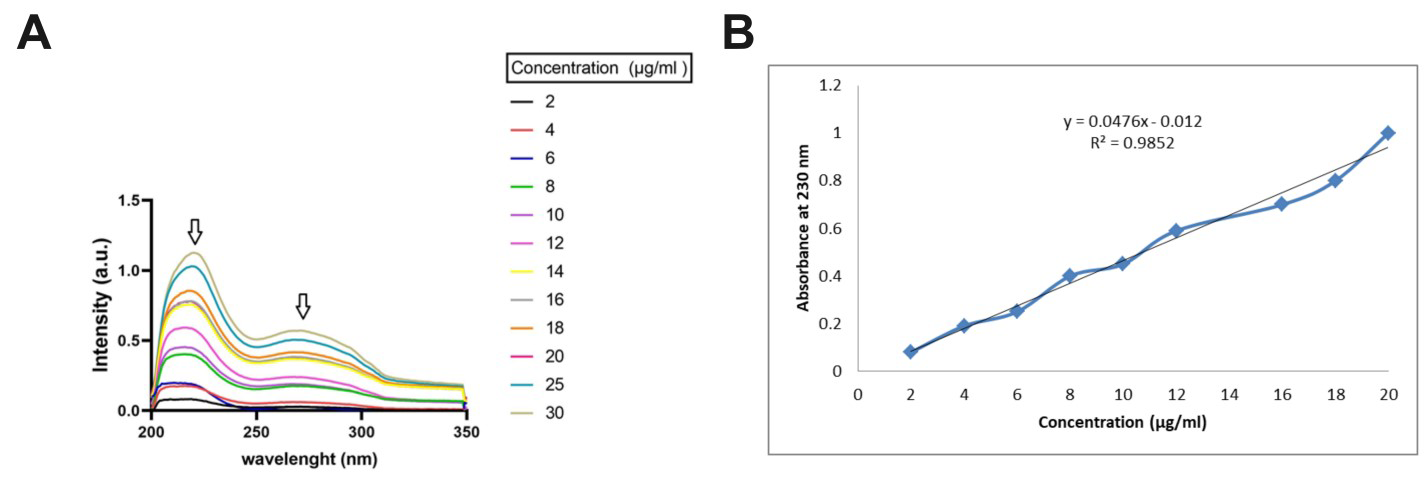

Supplement: S1 Fig — A: UV-Visible Absorption Spectrum of A-ghrelin at Different Concentrations, B: The resulting calibration curve of A-ghrelin illustrates the correlation between absorbance and concentration. (TIF) [file pone.0327235.s001.tif]
